# The impact of epigenetic landscape on ovarian cells in infertile older women undergoing IVF procedures

**DOI:** 10.1186/s13148-023-01490-0

**Published:** 2023-05-04

**Authors:** Giulia Sgueglia, Salvatore Longobardi, Domenico Valerio, Maria Rosaria Campitiello, Nicola Colacurci, Cinzia Di Pietro, Rosalia Battaglia, Thomas D’Hooghe, Lucia Altucci, Carmela Dell’Aversana

**Affiliations:** 1 Department of Precision Medicine, University of Campania ‘Luigi Vanvitelli’, Programma di Epigenetica Medica, Azienda Ospedaliera Universitaria, Naples, Italy; 2 Merck KGaA, 64293 Darmstadt, Germany; 3 Outpatient Fertility Unit, University of Campania ‘Luigi Vanvitelli’, 80138 Naples, Italy; 4 Department of Obstetrics and Gynecology and Physiopathology of Human Reproduction, ASL Salerno, Salerno, Italy; 5 Department of Woman, Child and General and Special Surgery, University of Campania ‘Luigi Vanvitelli’, 80138 Naples, Italy; 6 Department of Biomedical and Biotechnological Sciences, Section of Biology and Genetics “Giovanni Sichel”, University of Catania, 95123 Catania, CT Italy; 7 BIOGEM, Ariano Irpino, Italy; 8 Institute of Experimental Endocrinology and Oncology ‘Gaetano Salvatore’ (IEOS)-National Research Council (CNR), Naples, Italy

**Keywords:** Epigenetics, Fertility, IVF, Reproduction

## Abstract

The constant decline in fertility and older reproductive age is the major cause of low clinical pregnancy rates in industrialised countries. Epigenetic mechanisms impact on proper embryonic development in women undergoing in vitro fertilisation (IVF) protocols. Here, we describe the main epigenetic modifications that may influence female reproduction and could affect IVF success.

**Graphical Abstract:**

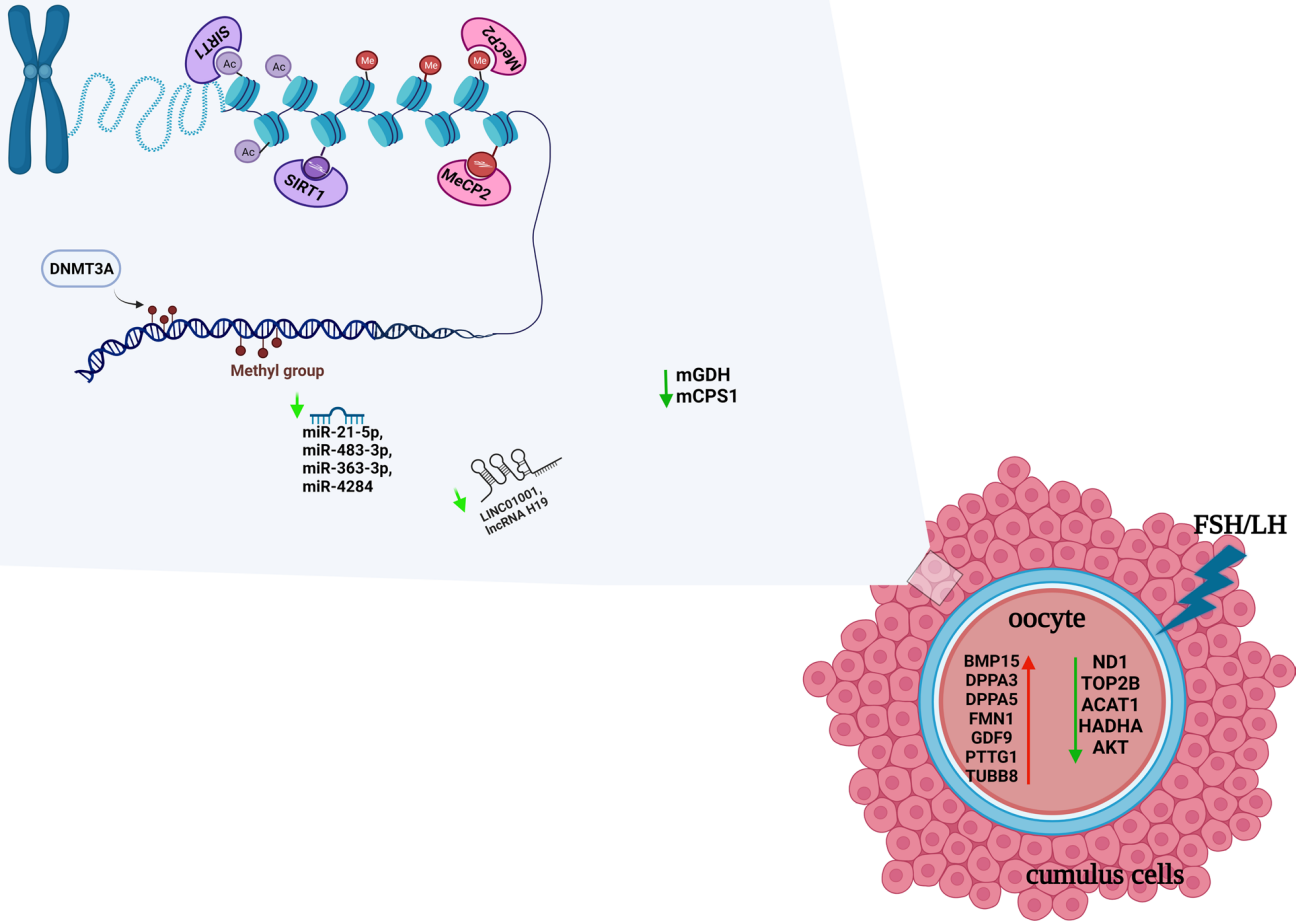

## Introduction

The evident decline in human fertility seen across industrialised countries [1, 2] is increasing the demand for medically assisted reproduction (MAR) procedures including *in vitro* fertilisation (IVF) [3]. One reason for the decrease in fertility is the trend towards postponing parenthood until the mid-30s and beyond [3]. Advanced reproductive age is also the main cause of the low rates of clinical pregnancy observed in older women undergoing MAR [4]. Most studies focus attention mainly on the alteration of female reproductive system; nevertheless, innovative studies have shown that even the paternal heritage can negatively influence the *in vitro* fertilisation [5–7].

The female reproductive system is the first organ system to age; senescence in female reproduction occurs decades before the functional decline in other organs. Reproductive ageing is associated with a reduction in oocyte quantity and quality, both of which contribute to the increased incidence of infertility and miscarriage reported for older women as reported by the American College of Obstetricians and Gynecologists (ACOG) as well as the possible heightened risk of congenital anomalies reported in their infants [8].

Although advances in IVF have enabled several forms of infertility to be treated, implantation and pregnancy rates following embryo transfer remain low in women aged >35 years; this is largely a result of the above-mentioned decline in the quality and quantity of oocytes that occurs over time [9]. Ageing reduces the ovarian reserve, decreases oocyte and embryo competence, and reduces the chances of success in MAR by increasing the aneuploidy rate and reducing levels of mitochondrial activity [10]. Consequently, to improve reproductive outcomes among older women, it is crucial to identify additional biomarkers, in addition to the few already used ones such as Connexin (Cx43) [11] BCL2 Associated X (BAX), steroidogenic acute regulatory protein (STAR), and prostaglandin-endoperoxide synthase 2 (PTGS2) [12] that characterise their ‘oocyte phenotype’ and predict the efficacy and outcomes of IVF treatment.

Epigenetic alterations, considered cellular and molecular hallmarks, drive the modified cellular functions that are characteristic of ageing [13]. Epigenetic mechanisms refer to heritable sets of changes, such as DNA cytosine methylation, histone modifications, and non-coding RNAs, able to instruct the cells to read a DNA sequence and rearrange the chromatin structure, thus finely orchestrating gene expression. Epigenetic control defines cell and tissue identity and regulates life-sustaining cellular functions. Twin studies unequivocally demonstrate that genetic factors account for 20–30% of variations in human lifespan, with the remaining 70–80% attributed to random events or environmental factors [14]. Genome-wide analyses have highlighted the crucial role of epigenetic mechanisms from the onset of ageing; specifically, ageing is associated with a reduction in global genomic methylation, including tissue-specific genes and CpG-poor promoters [15–17].

Although a strong direct correlation between altered methylation state, due to epigenetic modifications, and assisted reproduction implantation has not yet been demonstrated, many studies hypothesise that the DNA abnormal methylation patterns in the oocytes could negatively influence the reproductive potential [18–20]. Therefore, several new biological age predictors have recently been introduced, including epigenetic clocks. Although the most famous model is the Horvath clock, this predictor can hardly be used for tissue undergoing hormonal changes. Studies show that epigenetic aging of cumulus oophorous cells does not go at the same rate with woman chronological age; for this reason, a new model called Granulosa Cell clock has been developed, even if it still has a quite high DNA methylation error rates [21].

Interestingly, nutrients taken in the diet can also be crucial in the modulation of aging; in fact, has been demonstrated that for example, an incorrect intake of folate and homocysteine can significantly alter DNA methylation (in this case leading to hypermethylation) and subsequent epigenetic reprogramming of cell [22–25].

‘Omics’ technologies have identified hundreds of genes that play functional roles during the early stages of reproduction, influencing oocyte growth/maturation, endometrial receptivity, embryo development, and embryo-endometrial signalling; likewise, omics-based research has identified several potential biomarkers [26]. Nevertheless, little is known about upstream molecular mechanisms involved in the expression of these genes. Epigenetics is emerging as a ‘hot’ new field for investigating diagnostic, prognostic, and therapeutic techniques in reproductive medicine [27]. Transcriptional and post-transcriptional gene regulation and cell-to-cell communication in cumulus–oocyte complexes (COCs) are reported to coordinate critical aspects of epigenetic, transcriptional, and proteomic rearrangement [28]. Similarly, microRNAs (miRNAs) could be implicated in regulating oocyte–COC crosstalk [29].

To better understand the role of epigenetic modifications in oocyte ageing and human infertility, several studies have investigated biomarkers involved in crucial regulatory cell networks underlying oocyte competence and ageing [30–33]. The impact of gonadotropin stimulation as a potential epigenetic modulator in the physiology of COCs has also been explored [34, 35]. In this review, we describe our current knowledge and key scientific findings relating to the epigenetic landscape in aged women undergoing IVF and its association with ovarian stimulation protocols.

## The role of genomic integrity and histone modifications

With advancing age, the integrity of the genome is gradually lost, resulting in changes in chromatin accessibility. The chemical perturbations that can result in DNA lesions, genomic mutations, and transcriptional changes increase with age. Epigenetic modifications affect gene transcriptional regulation and very often involve histone phosphorylation, acetylation, and methylation. Histone acetylation and deacetylation are catalysed by enzymes known as histone acetyltransferases (HATs) and histone deacetylases (HDACs), which can transfer or remove one of the acetyl groups from or to lysine residues at histone H3 and H4 [36]. HATs are classified into type A and type B superfamilies, while HDACs are divided into class I, II, III and IV. Class I includes HDAC1, 2, 3, 8, and while class II, the only class of enzymes that can shuttle between nucleus and cytoplasm, includes HDAC4, 5, 6, 7, 9, and 10. Class III HDACs, also called sirtuins (SIRTs), require NAD^+^ for the deacetylation reaction. Class IV includes only HDAC11 [37].

The main class of epigenetic enzymes involved in oocyte ageing are SIRTs. A recent study also describes SIRTs as protectors of germ cells against oxidative stress [38]. Some SIRTs (SIRT1, 2, 3, and 6) are involved in chromatin regulation via acetylation of K16 and K9 on histone H4 and H3, respectively [39]. The SIRT family member most involved in fertility processes is SIRT1, which is mainly nuclear and participates in heterochromatin formation [40, 41]. SIRT1 plays a vital role in sustaining genomic integrity by maintaining the normal chromatin state of cells, thus protecting cells from oxidative stress, promoting DNA stability, and decreasing various age-related disorders such as neurodegenerative diseases, metabolic abnormalities, and cancer [39]. SIRT1, 2, 3 are known to rescue abnormal mitochondrial distribution in post-ovulatory aged oocytes, and SIRT1 is specifically involved in the modulation of mitochondrial functions, probably through the regulation of reactive oxygen species (ROS) production [42]. Another way in which mitochondrial dysfunction affects epigenetic changes in oocyte maturation is through the production of ROS, shown to act on H4K12 acetylation levels in porcine oocytes [43]. Dysregulation of ATP synthesis resulting from epigenetic modifications can also affect correct oocyte maturation, as the aberrant synthesis of ATP by mitochondria affects the production of S-Adenosyl methionine, a common donor of methyl groups for DNA methylation [44].

SIRT1 activity increases with age in reproductive cumulus cells and is triggered as a rescue mechanism to prevent cell senescence and ameliorate DNA damage following oxidative stress [31]. SIRT1 can also facilitate recruitment of primordial follicles (PFs) by directly modulating Akt and mammalian target of rapamycin (mTOR) [45]. Overall, the epigenetic functions exhibited by SIRT1 in human reproductive cells underpin the rationale for exploring the use of SIRT1 activator molecules, including resveratrol (RSV) and synthetic molecules, in MAR [46, 47]. The importance of SIRT1 in the ovary was also demonstrated by the use of RSV, a SIRT1 activator able to upregulate its expression in ovaries [48]. A recent study reported a potential therapeutic effect of RSV in improving ovarian function [30]. Considering the role of SIRT1 in PF activation, RSV-induced *in vitro* activation could therefore be an important strategy to improve procedures for the clinical treatment of infertility [45].

SIRT3 and SIRT5, specifically located in mitochondria, are able to regulate the mitochondrial metabolic regulator glutamate dehydrogenase (GDH) in both granulosa and CCs as well as the activity of carbamoyl phosphate synthetase (CPS1), involved in the urea cycle, thus altering the microenvironment around the oocyte in women with little ovarian reserve or in advanced maternal age [49].

In 2018, Valerio et al. first described the role of SIRT1 in telomere homeostasis of CCs. The authors observed a significant increase in SIRT1 messenger RNA (mRNA) levels in patients over 38 years old and found a positive correlation between SIRT1 mRNA levels and telomere length [31]. High mRNA levels of SIRT1 in CCs associated with the detection of SIRT1 finding highlight the relationship between high SIRT1 levels and total antioxidant levels [50]. In 2019, Zhang et al., showed that SIRT1 activates mouse PFs independently of its deacetylase activity [45]. Furthermore, SIRT1 was found expressed in pre-granulosa cells (pGCs) and oocytes, and its expression increased during PF activation, showing that SIRT1 promotes the differentiation of pGCs into granulosa cells, while in oocytes, it activates PTEN-PI3K-Akt signalling. In 2021, Szymanska et al. showed that SIRT1 can inhibit endothelin-2 (EDN2) expression in human granulosa-lutein cells (hGLCs) via hypoxia inducible factor 1 alpha (HIF1A) [46]. EDN2 expression in hGLCs was previously described as being dependent on HIF1A, and since HIF1A expression depends on SIRT1, HIF1A is suppressed by RSV (and other SIRT1 activators), while hypoxia reduces SIRT1 levels (mutual inhibition). In addition, SIRT1 was able to repress EDN2 expression by binding the EDN2 promoter and to decrease histone H3 acetylation. In 2019, Yun-Jung Choi et al. described the effect of tubastatin A (TubA) on mouse oocytes. TubA is an HDAC and SIRT inhibitor and triggers inadequate histone deacetylation leading to chromatin perturbation. TubA is responsible for a reduction in SIRT2 mRNA levels, which in turn leads to hyperacetylation of alpha-tubulin and failure of spindle or chromosome organisation [51]. Another tricky damage caused by aberrant acetylation in the oocyte maturation is aneuploidy [33], always related to the mother's elderly [52].

## DNA methylation imbalance

One of the main factors associated with a fertilisation failure is the reduced quality of oocytes in older women, often correlated with an alteration in nuclear and mitochondrial DNA [53]. It is known, for example, that the overall level of DNA demethylation in proliferating ovarian granulosa cells changes during mouse follicular development.

In granulosa cells and CCs, abnormal DNA methylation on critical gene promoters appears to adversely affect ovarian function in older women. Furthermore, the microenvironment can influence the state of DNA methylation and therefore the proper oocyte functionality. It seems, in fact, that some factors secreted by oocytes can markedly modulate epigenetic changes in terms of de/methylation in this type of cells. Some infections, such as bacterial infections of the female genital tract, can also easily alter the DNA methylation status of granulosa cells, thus affecting fertility [54].

As with nuclear DNA, mitochondrial DNA (mtDNA) can also undergo epigenetic changes including methylation. However, investigations into this type of modification are very challenging, and conflicting findings are reported in the literature on the correlation between mtDNA methylation levels and right oocyte development and maturation [29, 39]. One study reports that epigenetic modifications can be influenced by both external and internal factors and that these changes, although reversible, are inherited by the daughter cells [39]. The level of mitochondrial methylation seems to differ in granulosa cells and in oocytes as well as during oocyte maturation, and blastocysts display the highest levels [55]. It was also shown that an adverse external environment can modify the mtDNA methylation state in oocyte and blastocyst cells, suggesting its role not only as a qualitative indication of the oocyte itself, but also as a marker of subsequent embryonic development. This different methylation state could account for achieving fertilisation with increasing age [55]. Conversely, another study found no methylation at mtDNA level during mouse oocyte maturation, ageing, and early embryo development [44]. In some pathological conditions, such as endometriosis, CCs express low levels of CYP19A1, a cytochrome P450 aromatase responsible for oestrogen biosynthesis. This aberrant expression is due to a different modulation of its regulatory regions by MeCP2, a methyl-binding protein involved in DNA methylation. The effect of this altered regulation is the impairment of follicular steroidogenesis, resulting in poor condition of the oocytes [56].

Hormones may also be responsible for epigenetic alterations in the reproductive tract. For example, oestrogen replacement therapy in menopausal women reduces total homocysteine in cells, resulting in increased methylation of mononuclear cell genomic DNA. In the rodent brain, oestrogens are responsible for modulating methylation on the promoter of oestrogen receptor alpha [32].

With the advancement of single-cell techniques and high-throughput sequencing platforms, single-cell RNA sequencing (scRNA-seq) has emerged as an essential technology for understanding tissue and organ systems at cellular resolution [57]. Comparison of the transcriptome at single cells level from younger and older patients undergoing IVF treatments may help understand the molecular mechanisms of oocyte quality and embryo development. The first study highlighted differences in gene expression between oocytes deriving from women of different ages. In particular, older oocytes showed a strong deregulation of ND1, TOP2B (chromatin structure gene), and two genes associated with DNA damage repair, RAD50 and RAD17 [57]. One of the most important data emerging from the scRNA-seq study is the difference in gene expression between oocytes matured in vitro and in vivo. Indeed, in vitro environment can lead to a decline in metabolism activity of CoA-related enzymes, such as ACAT1 and HADHA, while gene changes have been emphasised in oocytes from polycystic ovarian syndrome patients, especially associated with mitochondrial function as COX6B1, COX8A, COX4l1, and NDUFB9 [58, 59].

Finally, a new single cell multi-omics sequencing method has been developed, used for oocytes, called scCOOL-seq with which both DNA methylation and chromatin accessibility can be studied in the same cell. These results showed, for instance, that mature oocytes have a lower DNA methylation than ovarian somatic cells, conversely granulosa cells and stroma cells, had a similar level of global DNA methylation. The differentially methylated regions confirmed single-cell RNA-seq performed by Zhang et al., wherein BMP15, DPPA3, DPPA5, FMN1, GDF9, PTTG1 and TUBB8 genes showed relatively higher expression levels in mature human oocytes. Considering instead the common epigenetic interactors, it has been shown that both DNMT1 and DNMT3A are expressed in growing oocytes and mature oocytes, while DNMT3B and UHRF1 are mainly expressed only in mature oocytes [60].

## miRNA and long non-coding RNA recruitment

MiRNAs are small non-coding RNAs (20–25 nucleotides) mainly involved in regulating the expression of more than 30% of genes [61]. They are associated with specific processes such as cellular and early embryo development [62]. miRNAs can also act as epigenetic modulators and are regulated by epigenetic modifications such as DNA methylation, and RNA and histone modifications [63]. Perturbation in their expression levels has a significant correlation with many human diseases, including cancer, neurodegenerative diseases, and metabolic disorders [64]. Since they are also found in extracellular vesicles of body fluids, current studies have shifted their focus to investigate the use of circulating miRNAs as non-invasive biomarkers for diagnostic, predictive, and prognostic purposes and as potential new tools to interfere with the molecular mechanisms of various pathologies [65].

A recently recognised class of miRNAs called epi-miRNAs are able to control the expression of key epigenetic enzymes involved in chromatin remodelling, including DNA methyltransferases (DNMTs), HDACs, histone methyltransferases (HMTs), and ten-eleven translocation enzymes (TETs) [66, 67]. For example, miR-29b directly downregulates the expression of DNMT3A and DNMT3B and indirectly that of DNMT1 [67, 68].

The importance of miRNAs in reproduction biology is becoming increasingly recognised. Several studies have investigated miRNA function in ovarian follicle components and described their essential role in folliculogenesis, oocyte growth, and meiosis resumption. Specifically, miRNAs have emerged as important regulators of oogenesis, spermatogenesis, fertilisation, embryogenesis, and cumulus–oocyte crosstalk [29, 69–74]. MiRNome analysis has in fact been performed in endometrial, myometrial, cervical, and ovarian tissue samples [75–78]. The altered regulation of miRNA expression has been linked to several reproductive disorders, oocyte developmental abnormalities, and fertilisation failure [79, 80]. Furthermore, a correlation between changes in miRNA expression, oocyte ageing, and epigenetic modifications is also reported [75, 81, 82]. For example, a study using an *in vivo* mouse model found that the upregulation of miR-29a-3p and miR-203a-3p in old oocytes is inversely correlated with the downregulation of DNMT3A and DNMT3B [75]. miR-29b inhibition increased DNA methylation levels of the global genome by upregulating DNMT3A/B and TET1 and downregulating TET2/3 during porcine early embryo development [83]. Aberrations in miRNA expression, together with alterations of DNA methylation patterns, may contribute to compromised quality oocytes in older reproductive women that could be inherited by the embryo [32] and could therefore also drive human idiopathic infertility [27].

To identify non-invasive molecular markers of oocyte quality, cell-free miRNA expression was investigated in follicular fluid; exosomal miRNAs seem to be critically implicated in follicle development and oocyte maturation in human and porcine follicular fluid [84–86]. Recently, changes in miRNA expression profiles in human follicular fluid of women of older reproductive age have also been described [87, 88] and shown to influence processes related to oocyte maturation, stress responses, and vesicle secretion [82]. Specifically, 13 miRNAs influencing mitochondrial proteins (called mito-miRNAs) were recently reported to be differently expressed and regulated by RSV in follicular fluid derived from aged women with a poor ovarian reserve [89]. A better understanding of molecular mechanisms regulating gene expression, including the role of miRNAs, and a greater insight into other transcriptional and proteomic messengers in the extracellular microenvironment have highlighted the importance of cell-to-cell communication and the possibility of translating this approach to the clinic. The identification of new non-invasive biomarkers of oocyte quality and embryo competence and the development of novel therapeutics could improve reproductive outcomes in aged women.

Previous studies found that specific miRNAs target key ovarian players such as PGR, CYP19A1, and FSHR, which act as paracrine factors in the crosstalk between oocytes and CCs [69, 90]. In older women undergoing IVF, significantly higher miRNA levels were recorded in those receiving recombinant follicle-stimulating hormone (r-FSH+r-LH) than in those treated with r-FSH alone [20]. These findings confirm that a woman’s age is an independent factor affecting miRNA expression in CCs and further highlight the fact that gonadotropin treatment might impact both the expression of follicular miRNA and the efficacy of IVF. In infertile older women undergoing IVF, integrating r-LH in a r-FSH gonadotropin protocol appeared to provide a more effective level of epigenetic remodelling in CCs (in terms of miRNA content and expression profiling) than with r-FSH alone [35].

Long non-coding RNAs (lncRNAs) are heterogeneous RNA molecules longer than 200 nucleotides [91]. LncRNAs can modulate gene expression at different levels by controlling chromatin remodelling, regulating the assembly and function of nuclear bodies, and altering the stability and translation of cytoplasmic mRNAs; by interacting with mRNAs and miRNAs, they are also able to create a dynamic regulatory network, acting as competing endogenous RNAs (ceRNAs) [92]. LncRNA activity influences cell physiology and some lncRNAs has been demonstrated involved in different pathologies. Using an RNA sequencing approach, Jiao et al. identified 1583 lncRNAs in human follicular fluid and found that some were differentially expressed in healthy women compared to women affected by polycystic ovary syndrome [93]. LncRNAs have also been identified in human oocytes and in CCs, and metaphase II (MII) oocyte lncRNAs may be involved in chromatin remodelling, cell pluripotency, and in driving early embryonic development [74]. In contrast, CC lncRNAs were found co-expressed with genes involved in apoptosis and in extracellular matrix-related functions. Additionally, six lncRNAs were identified as downregulated in CCs from women of advanced reproductive age [94]. Despite clear evidence that lncRNAs are expressed in the ovarian follicle and that their altered expression is associated with reproductive disorders and ageing, their biological role, especially in humans, has only been described in very few cases. In mice, the lncRNA *Neat1* seems to be required for corpus luteum formation and the establishment of pregnancy, as *Neat1* knockout mice failed to become pregnant despite normal ovulation [95]. The lncRNA *H19* is part of a highly conserved imprinted gene cluster involved in embryo development [96]. *H19* downregulation in serum and CCs was recently shown to be closely associated with diminished ovarian reserve measured by decreased anti-müllerian hormone levels and reduced oocyte recovery [96].

In order to understand the specific function of lncRNAs in the follicle microenvironment, a recent study investigated lncRNAs downregulated in CCs from women of advanced reproductive age and mapped the ceRNA networks involving differentially expressed lncRNAs, miRNA interactors, and their mRNA target genes [97]. Some of the downregulated lncRNAs were found to be part of three ceRNA networks that can act on PI3K-Akt, FOXO, and p53 signalling pathways. These pathways are involved in different stages of ovarian follicle development and are known to control cellular growth and proliferation, oxidative stress, cellular senescence, and apoptosis. Alterations in lncRNA regulation have been extensively described in reproductive ageing [78]. LncRNA downregulation may in turn lead to the downregulation of specific mRNAs encoding key proteins associated with follicular maturation such as PTEN and SIRT1 [97]. Specifically, downregulation of *H19* could increase the expression of its interactors, miR-93-5p and miR-193a-3p, and consequently decrease the levels of PTEN, a validated target of the two miRNAs. Similarly, the increase in miR-138-5p expression mediated by downregulation of *H19* could cause the downregulation of SIRT1. Interestingly, PTEN and SIRT1 are components of the ceRNA network involving the little-known lncRNA long intergenic non-protein coding RNA 1001 (LINC01001) found downregulated in CCs from women of advanced reproductive age [97]. The identification of ceRNA networks dysregulated in CCs of MII oocytes from advanced maternal age women could represent a promising tool to evaluate the efficacy of different stimulation protocols. CC transcriptomic analysis and ceRNA network studies may allow us to discover potential candidate biomarkers and therapeutic targets in CCs under pathological conditions.

## Conclusions

The main reason for the continuing decline in fertility is the tendency to postpone parenthood until and beyond the age of 30, mainly for socio-economic reasons. Postponing pregnancy involves a higher risk of infertility due to the physiological deterioration of the female reproductive system, especially in terms of the ovules available for correct and natural fertilisation. IVF is an assisted reproduction technique which, as the name suggests, allows the fertilisation of an ovum by a sperm in a laboratory setting. After successful fertilisation, the zygote can be transplanted into the woman’s uterus for the normal continuation of pregnancy. Despite now being a widely used technique, some studies report a higher prevalence of perinatal problems in humans, and findings obtained from animal experiments raise concerns about the occurrence of epigenetic abnormalities. Numerous animal models and both retrospective and follow-up studies on infants born from assisted reproductive technology procedures have shown an increased risk of epigenetic errors, especially those affecting imprinted loci [98]. It is therefore crucial to obtain a comprehensive understanding of the mechanisms underlying epigenetic changes associated with age and the factors that could interfere with or mitigate these changes [99] (Table 1). One of the main parameters to consider is the optimal state of the ovule at the time of collection; changes in the microenvironment (such as the hormones injected to stimulate ovulation) can alter normal ovular physiology. It would therefore be useful to design a methodology that is best able to ascertain the actual state of ‘well-being’ of the egg. Since the epigenetic state influences not only the oocyte physiology, but is also indicative of the conditions of the oocyte itself or of the cells which provide its nourishment (CCs) [13], specific epigenetic signatures could act as new markers able to predict oocyte phenotype and hence the effectiveness of IVF treatment. IVF has a huge impact on infertile couples and society at large, and greater efforts are needed to progress this technique while minimising unnecessary undesirable effects by sharing and discussing current knowledge on the epigenetic mechanisms involved in clinical infertility treatments.

**Table 1:** List of epigenetic modifiers involved in fertility process.

Epigenetic player	Expression level	Target (gene or relative pathway)	Cell/Tissue	References
SIRT1/2/3	↑	H3K9 acetylation	Post-ovulatory aged oocytes	[31]
SIRT1	↑	Telomere homeostasis	Cumulus cells	[20]
SIRT1	↑	Akt; mTOR	Primordial follicles	[34]
SIRT3 and SIRT5	↑	Urea cycle	Granulosa and CCs	[38]
SIRT1	↑	EDN2	Granulosa-lutein cells (hGLCs)	[35]
MeCP2	↑	CYP19A1	CCs	[44]
miR-29a-3p	↑	DNMT3A	Old oocytes	[59]
miR-203a-3p	↑	DNMT3B	Old oocytes	[59]
miR-29b	↑	DNMT3A/B and TET1	Porcine early embryo development	[67]
mito-miRNAs (miR-19a-3p, miR-30a-5p, miR-125b-5p, miR-132-3p and miR-660-5p)	↓	SIRT1, TNF-a, AKT	Follicular fluid	[73]
miRNAs 483-3p, 363-3p, and 4284	↓	Transforming growth factor (TGF)-β signalling pathway	Granulosa cells	[54]
lncRNA Neat1	↑	Luteal genes	Corpus luteum	[79]
lncRNA H19	↓	AMH (anti-müllerian hormone); miR-93-5p and miR-193a-3p	Serum and cumulus cells	[80, 81]
Long intergenic non-protein coding RNA 1001 (LINC01001)	↓	PTEN and SIRT1	CCs	[81]

In conclusion, further studies are required to better define the epigenetic modifications involved in affected developmental pathways and to fully investigate their clinical relevance as biomarkers for new treatments.

## Data Availability

Not applicable.
